# Supplementary material for: saRNA vaccine expressing membrane-anchored RBD elicits broad and durable immunity against SARS-CoV-2 variants of concern
Source: Nat Commun. 2023 May 19;14:2810. doi: 10.1038/s41467-023-38457-x (PMC10199074; doi:10.1038/s41467-023-38457-x)
Supplement: Supplementary file 1 — Supplementary Information [file 41467_2023_38457_MOESM1_ESM.pdf]

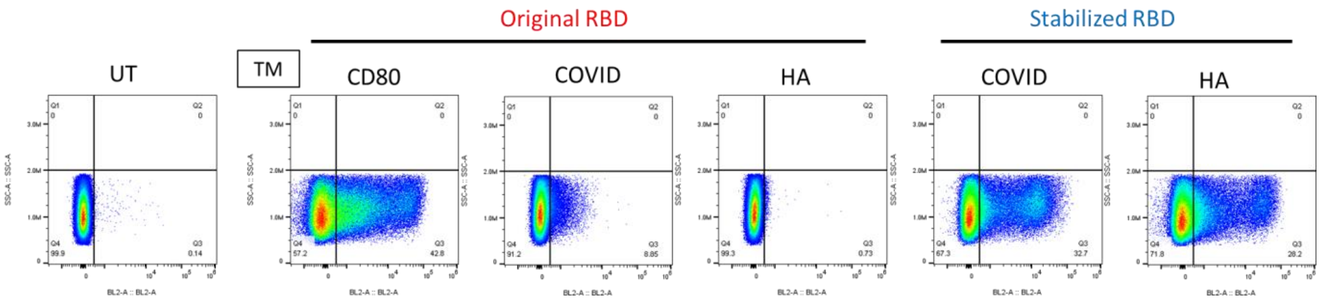

Supplementary Figure. 1| Comparison of Original RBD (330-521aa) and Stabilized RBD (327-531aa) with transmembrane for cell surface expression of FACS

293T cells were transfected with DNA encoding SARS-CoV-2 RBD with transmembrane. Cells were harvested 48 hours post transfection and evaluated for cell surface expression by FACS. Experiment was performed two times independently.

Supplementary Figure 2

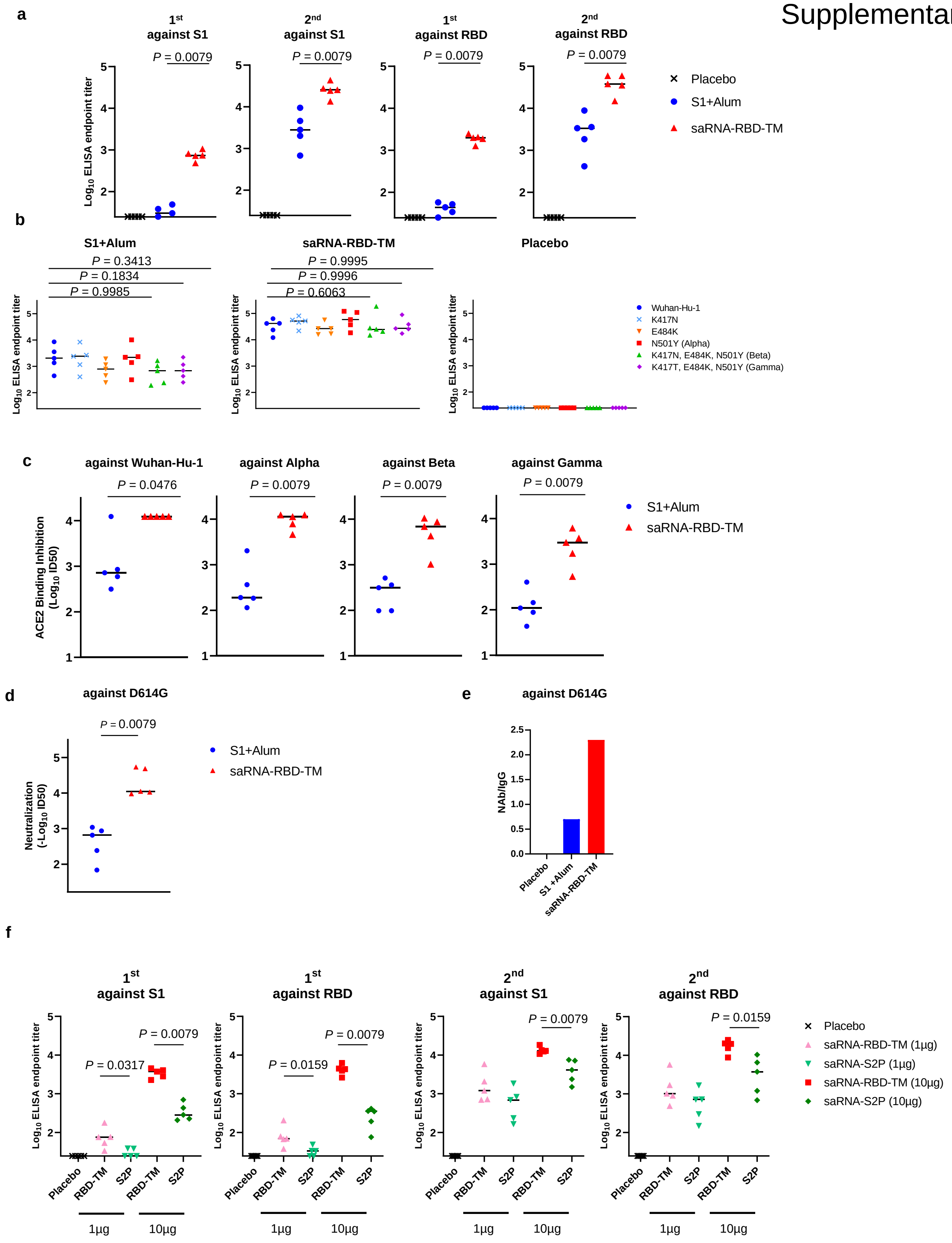

Supplementary Figure. 2| Comparison of immunogenicity of RBD and spike based vaccines in mice

a, BALB/c female mice (n = 5 per group) were immunized intramuscularly twice with 10 µg of S1 protein with alum, 10 µg of saRNA-LNP expressing SARS-CoV-2 Spike RBD with TM at a 4-week interval. Antibody titers of sera from the immunized mice were evaluated by ELISA against SARS-CoV-2 Spike S1 and RBD proteins at 4 weeks after 1st immunization (Day 28) and 2 weeks after 2nd (Day 42). Plots represent individual endpoint titers with median. Mann-Whitney test (two-tailed) was used to determine significance. Experiment was performed two times independently.

b, Antibody titers of sera (Day 56) from the immunized mice were evaluated by ELISA against variant SARS-CoV-2 Spike RBD antigens. Plot represents individual endpoint titers with medians . One-way ANOVA was used to determine significance. Experiment was performed two times independently.

c, The ACE2 and RBD binding inhibition titer of sera from the immunized mice at Day 56 were measured. Plots indicate the $Log_{10}ID_{50}$ with medians. Mann-Whitney test (two-tailed) was used to determine significance. Experiment was performed two times independently.

d, SARS-CoV-2 Neutralization assays against D614G (B.1.1. isolate bearing D614G) was performed for evaluating immunized mice sera at Day 42. Plots indicate the – $Log_{10}ID_{50}$ with medians. Mann-Whitney test (two-tailed) was used to determine significance. Experiment was performed two times independently.

e, The ratio of neutralizing antibody against D614G and IgG in mouse were calculated using ID_{50} values from ELISA and Virus Neutralizing assay.

f, BALB/c female mice (n = 5 per group) were immunized intramuscularly twice with 1 µg or 10 µg of saRNA-LNP expressing SARS-CoV-2 Spike protein (S2P) or SARS-CoV-2 RBD with TM at a 3-week interval. Antibody titers of sera from the immunized mice were evaluated by ELISA against SARS-CoV-2 Spike S1 and RBD proteins at 3 weeks after 1st immunization (Day 21) and 2 weeks after 2nd (Day 35). Plots represent individual endpoint titers with median. Mann-Whitney test (two-tailed) was used to determine significance. Experiment was performed two times independently.

Supplementary Figure 3

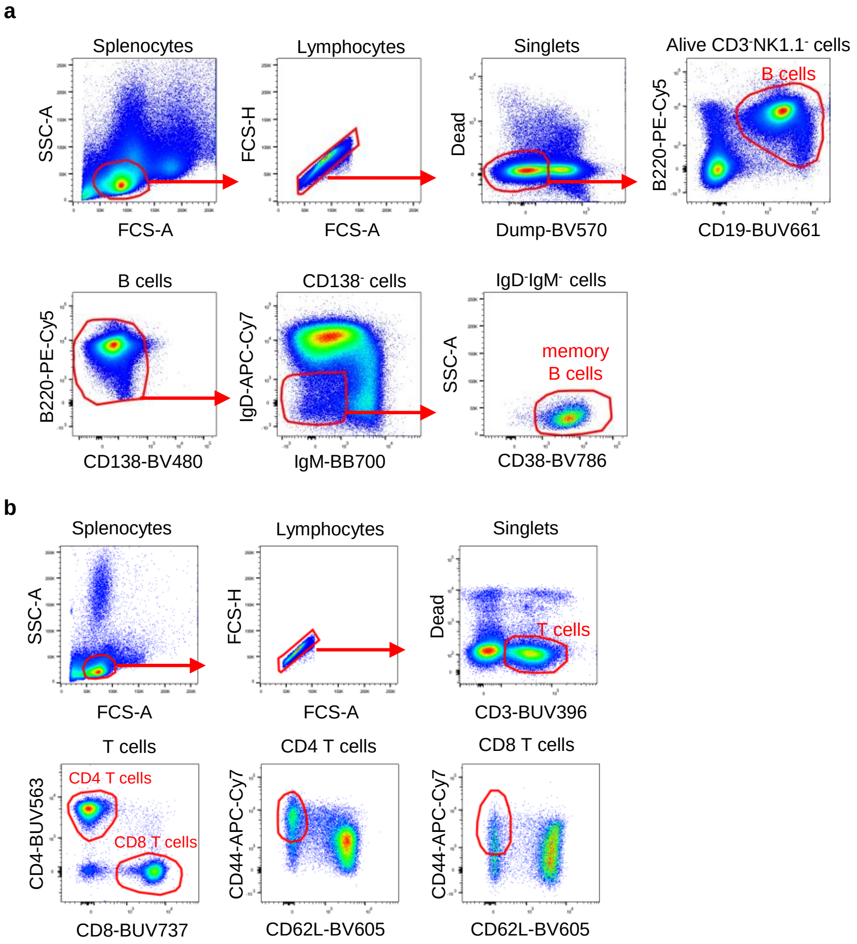

Supplementary Figure 3

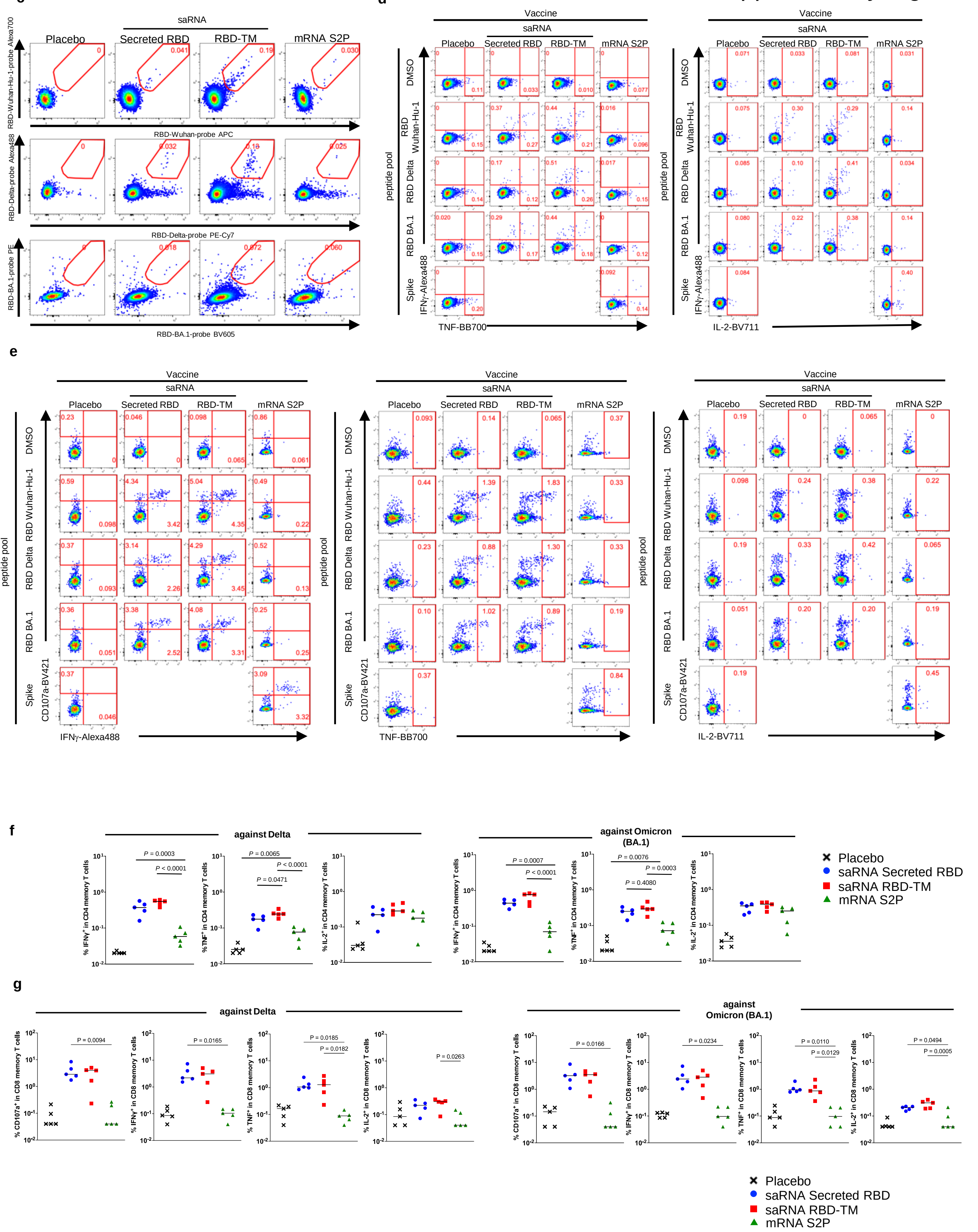

Supplementary Figure. 3| Immunogenicity of saRNA RBD vaccine and mRNA Spike vaccine in mice related to Fig. 2

BALB/c female mice (n = 5 per group) were immunized intramuscularly twice with a four week interval with 1 µg of saRNA-LNP expressing SARS-CoV-2 Spike variant RBD with or without TM or with 10 µg of mRNA-LNP expressing the Spike protein (S2P). All experiments were performed once.

a, After gating live single B cells, based on forward scatter area and height (FSC-A and -H), side scatter area (SSC-A), live/dead cell exclusion, $CD3^{+}NK1.1^{+}$ cell exclusion and CD19/B220 staining, we further gated the cells into $IgD^{-}IgM^{-}CD138$-$CD38^{+}$ cells as memory B cells.

b, After gating live single T cells, based on forward scatter area and height (FSC-A and -H), side scatter area (SSC-A), live/dead cell exclusion, and CD3 staining, we separated the cells into $CD4^{+}$ and $CD8^{+}$ T cells. Subsequently, $CD4^{+}$ and $CD8^{+}$ T cells were further divided into memory phenotypes based on the expression of CD62L and CD44.

c, Gating strategy for quantification of RBD-specific B cells. Plots were gating on $CD3^{-}NK1.1^{-}CD19^{+}B220^{+}IgD^{-}IgM^{-}CD138^{-}CD38^{+}$.

d, Gating strategy for quantification of RBD-specific CD4 T cells. Plots were gating on $CD3^{+}CD4^{+}CD62L^{-}CD44^{+}$.

e, Gating strategy for quantification of RBD-specific CD8 T cells. Plots were gating on $CD3^{+}CD8^{+}CD62L^{-}CD44^{+}$.

f, Dot plots representing the frequencies of IFN-γ, TNF, or IL-2-secreting $CD4^{+}$ T cells responding to RBD Delta (upper panels) or BA.1 (lower panels) peptides in $CD4^{+}$ total memory cells (. The lines show the medians. One-way ANOVA was used to determine significance.

g, Dot plots representing the frequencies of CD107a, IFN-γ, TNF, or IL-2-secreting $CD8^{+}$ T cells responding to RBD Delta (upper panels) or BA.1 (lower panels) peptides in $CD8^{+}$ total memory cells. The lines show the medians. One-way ANOVA was used to determine significance.

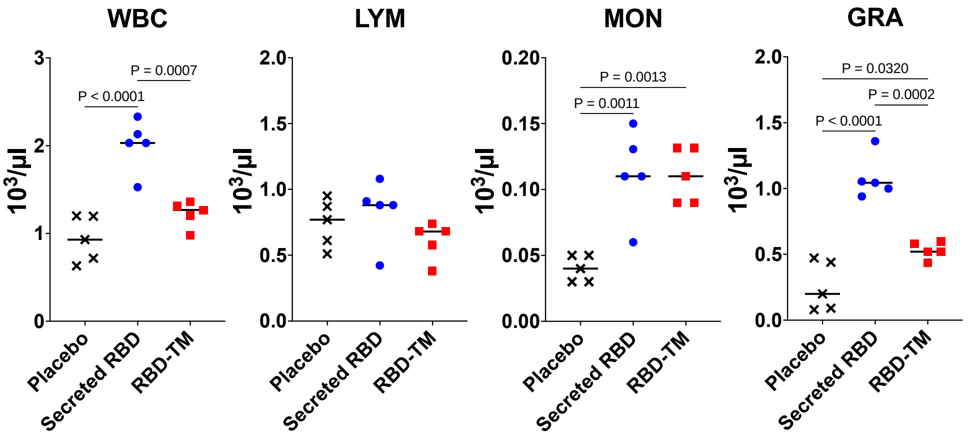

Supplementary Figure. 4| Blood cell counts in saRNA-vaccinated mice

BALB/c female mice (n = 5 per group) were immunized intramuscularly once with 1 µg of saRNA-LNP expressing SARS-CoV-2 Spike variant RBD with (RBD-TM) or without (Secreted RBD) HA TM. Dot plots represent the absolute cell counts for white blood cells (WBC), lymphocytes (LYM), monocytes (MON) and granulocytes (GRA) 8 hours after immunization. One-way ANOVA was used to determine significance. Experiment was performed once.

Supplementary Figure 5

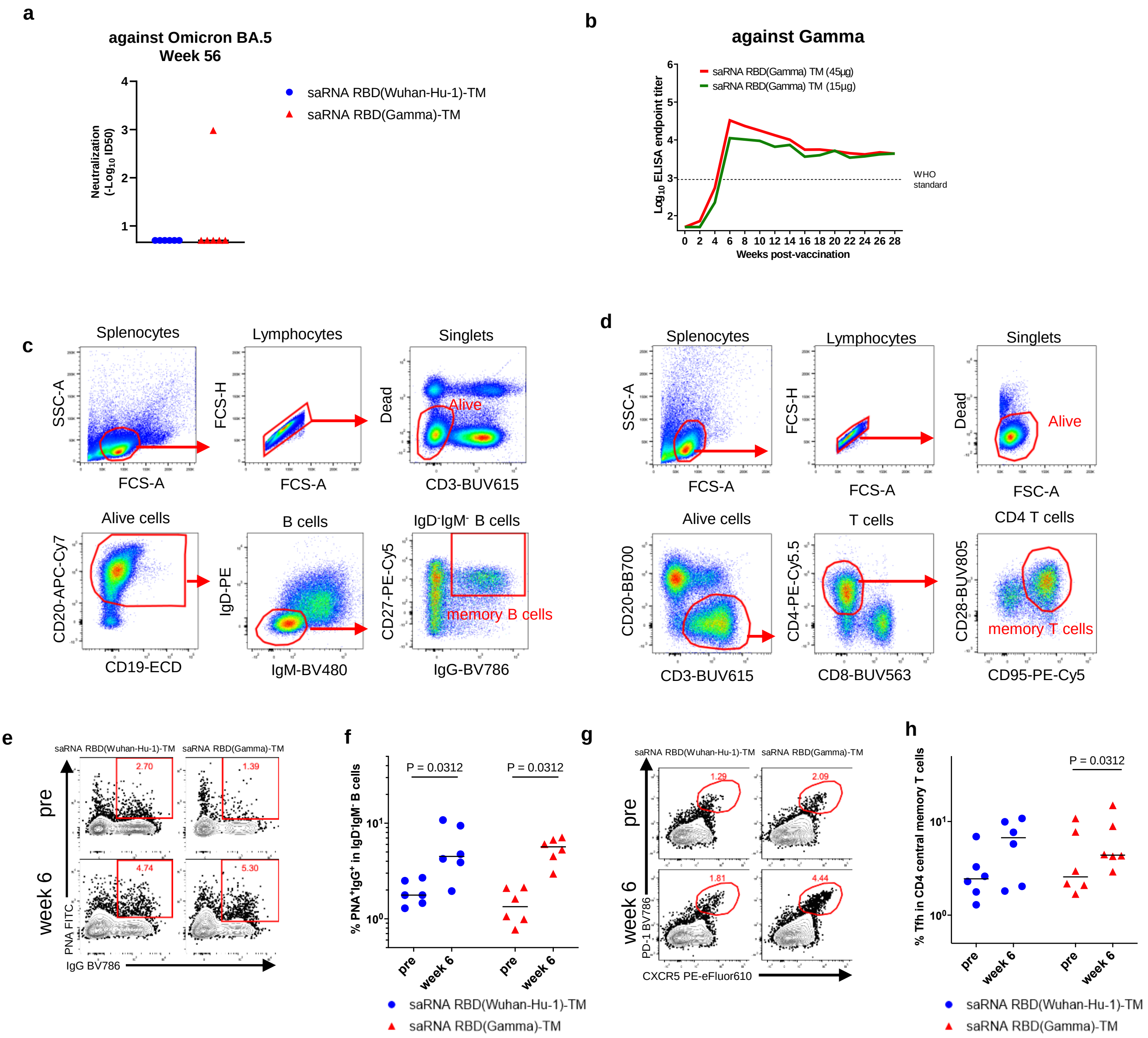

Supplementary Figure 5

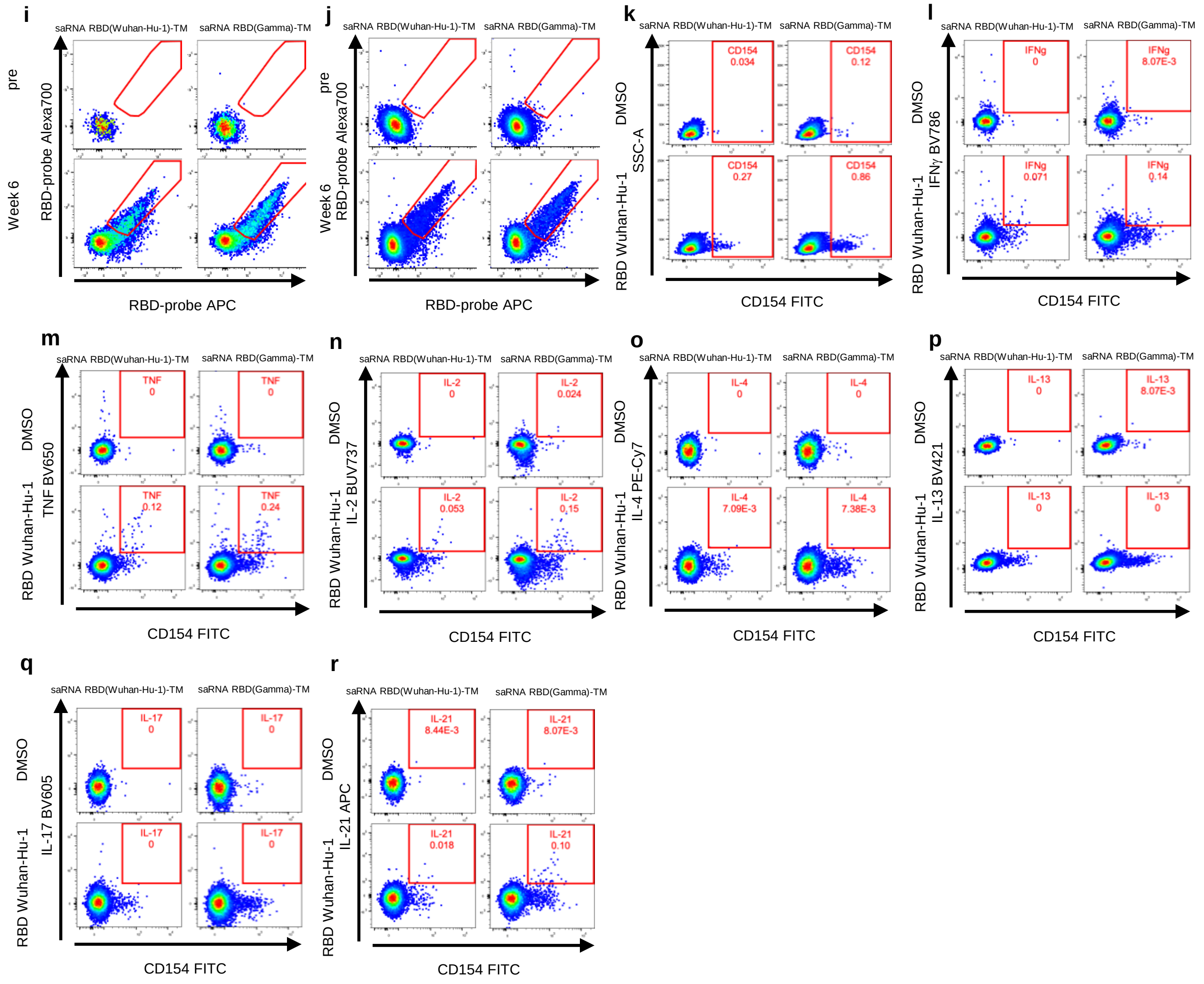

Supplementary Figure. 5| Immunogenicity of RBD vaccine in non-human primate model related to Fig.5

a, SARS-CoV-2 Neutralization assays against Omicron (BA.5) was performed for evaluating immunized sera at week 56. Plots indicate the – $Log_{10}ID_{50}$ with medians. Experiment was performed once.

b, Antibody titers of plasma from the immunized macaques (n = 6 per group) were evaluated by ELISA against SARS-CoV-2 Gamma RBD. Dot-dashed lines show the endpoint titers of WHO international Standard for anti-SARS-CoV-2 immunoglobulin, human (WHO standard, NIBSC code: 20/136).

c, After gating live single B cells, based on forward scatter area and height (FSC-A and -H), side scatter area (SSC-A), live/dead cell exclusion, $CD3^{+}$ cell exclusion and CD19/CD20 staining, we further gated the cells into $IgD^{-}IgM^{-}CD27^{+}IgG^{+}$ cells as memory B cells. Following experiments were performed once.

d, After gating live single T cells, based on forward scatter area and height (FSC-A and -H), side scatter area (SSC-A), live/dead cell exclusion, $CD20^{+}$ cell exclusion and CD3 staining, we separated the PBMCs into $CD4^{+}$ and $CD8^{+}$ T cells. Subsequently, $CD4^{+}$ T cells were further divided into memory phenotypes based on the expression of CD28 and CD95.

e, Gating strategy for quantification of germinal center (GC) B cells. GC B cells were defined as $CD20^{+}IgD^{-}IgM\text{-}PNA^{+}IgG^{+}$.

f, Dot plot represents the frequencies of GC B cells as a percentage of $IgD^{-}IgM^{-}$ B cells in a lymph node (n = 6 per group). The lines show the geometric means. *P*-values (two-sided) were calculated using the Wilcoxon matched-pairs signed rank test.

g, Gating strategy for quantification of Tfh in a lymph node. Tfh cells were defined as $CD20^{-}CD3^{+}CD4^{+}CD28^{+}CD95^{+}CXCR5^{+}PD\text{-}1^{+}$.

h, Dot plot represents the frequencies of Tfh cells as a percentage of CD4 central memory T cells in a lymph node (n = 6 per group). The lines show the geometric means. *P*-values (two-sided) were calculated using the Wilcoxon matched-pairs signed rank test.

i, j, Gating for quantification of RBD-specific B cells in memory B cells. Plots were gating on $CD20^{+}IgD^{-}IgM\text{-}CD27^{+}IgG^{+}$.

k-r, Gating for quantification of RBD-specific $CD4^{+}$ T cells. Plots were gating on $CD20^{-}CD3^{+}CD4^{+}CD28^{+}CD95^{+}$.

Supplementary Figure 6

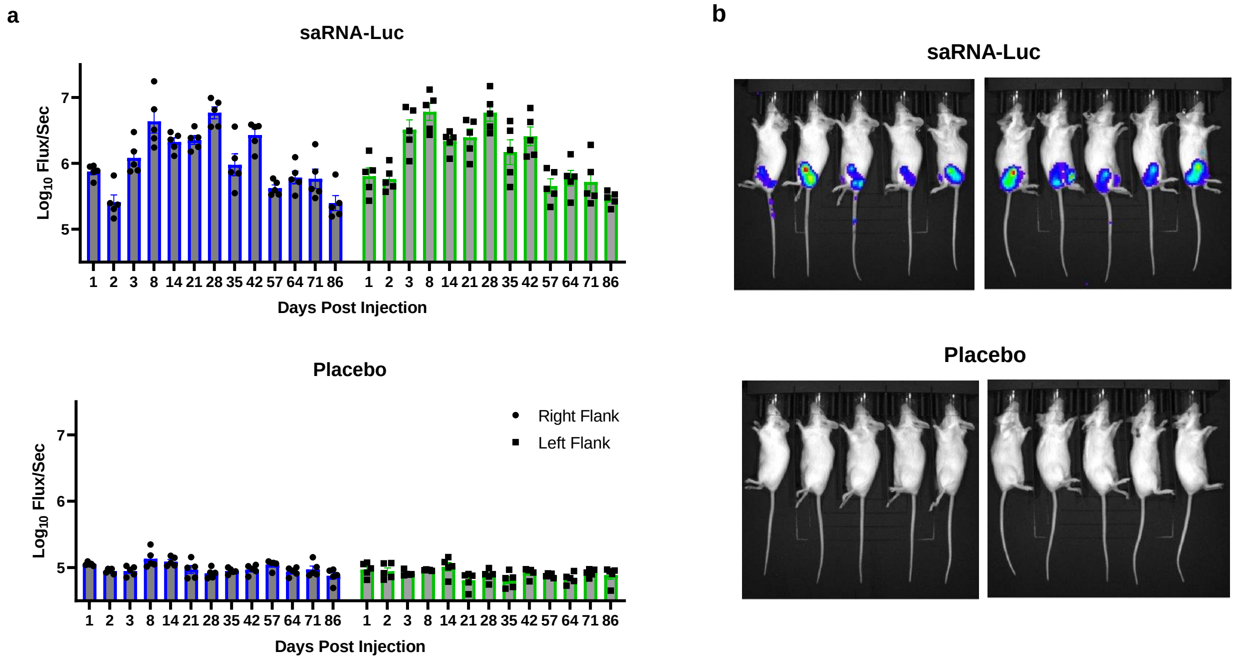

Supplementary Figure. 6 | Imaging of saRNA expressing Luciferase in mice

BALB/c female mice (n=5 per group) were injected intramuscularly once with 10 µg of saRNA-Luciferase-LNP or saRNA-RBD-TM-LNP (Placebo) on the right and left flank at Day 0. IVIS imaging was performed at Day 1, 2, 3, 8 and following every seven days until Day 86.

a, Plots represent individual Log_{10} Flux/Sec with mean $\pm$ SEM.

b, Image at 28 days post injection.
