# Supplementary material for: saRNA vaccine expressing membrane-anchored RBD elicits broad and durable immunity against SARS-CoV-2 variants of concern
Source: Nat Commun. 2023 May 19;14:2810. doi: 10.1038/s41467-023-38457-x (PMC10199074; doi:10.1038/s41467-023-38457-x)
Supplement: Supplementary file 3 — Reporting Summary [file 41467_2023_38457_MOESM3_ESM.pdf]

# nature portfolio

Corresponding author(s): Wataru Akahata, Takuya Yamamoto, Jonathan F. Smith

Last updated by author(s): Mar 31, 2023



# Reporting Summary

Nature Portfolio wishes to improve the reproducibility of the work that we publish. This form provides structure for consistency and transparency in reporting. For further information on Nature Portfolio policies, see our Editorial Policies and the Editorial Policy Checklist.

## Statistics

For all statistical analyses, confirm that the following items are present in the figure legend, table legend, main text, or Methods section.

| n/a | Confirmed | |
|---|---|---|
| ☐ | ☒ | The exact sample size (*n*) for each experimental group/condition, given as a discrete number and unit of measurement |
| ☐ | ☒ | A statement on whether measurements were taken from distinct samples or whether the same sample was measured repeatedly |
| ☐ | ☒ | The statistical test(s) used AND whether they are one- or two-sided<br>*Only common tests should be described solely by name; describe more complex techniques in the Methods section.* |
| ☒ | ☐ | A description of all covariates tested |
| ☐ | ☒ | A description of any assumptions or corrections, such as tests of normality and adjustment for multiple comparisons |
| ☐ | ☒ | A full description of the statistical parameters including central tendency (e.g. means) or other basic estimates (e.g. regression coefficient) AND variation (e.g. standard deviation) or associated estimates of uncertainty (e.g. confidence intervals) |
| ☐ | ☒ | For null hypothesis testing, the test statistic (e.g. *F*, *t*, *r*) with confidence intervals, effect sizes, degrees of freedom and *P* value noted<br>*Give P values as exact values whenever suitable.* |
| ☒ | ☐ | For Bayesian analysis, information on the choice of priors and Markov chain Monte Carlo settings |
| ☒ | ☐ | For hierarchical and complex designs, identification of the appropriate level for tests and full reporting of outcomes |
| ☒ | ☐ | Estimates of effect sizes (e.g. Cohen's *d*, Pearson's *r*), indicating how they were calculated |

*Our web collection on statistics for biologists contains articles on many of the points above.*

## Software and code

Policy information about availability of computer code

| | |
|---|---|
| Data collection | Initial raw data was collected using the Microsoft Excel (Office 365) software package. |
| Data analysis | FlowJo Software version 10.8.1. (BD, Ashland, OR) and Simplified Presentation of Incredibly Complex Evaluations version 6.1 provided by Dr. Mario Roederer (National Institutes of Health, Bethesda, MD) was used for analyzing flow cytometry data. Statistical analyses were conducted by GraphPad Prism 9.5.1 (GraphPad software, San Diego, CA). Image LabTM Software (Bio-Rad) was used for analysis of Western Blot images. |

For manuscripts utilizing custom algorithms or software that are central to the research but not yet described in published literature, software must be made available to editors and reviewers. We strongly encourage code deposition in a community repository (e.g. GitHub). See the Nature Portfolio guidelines for submitting code & software for further information.

## Data

Policy information about availability of data

All manuscripts must include a data availability statement. This statement should provide the following information, where applicable:

- Accession codes, unique identifiers, or web links for publicly available datasets
- A description of any restrictions on data availability
- For clinical datasets or third party data, please ensure that the statement adheres to our policy

The data that support this study are available from the corresponding authors upon request. The source data underlying Figures 1–5 have been provided as a Source Data file.

## Human research participants

Policy information about studies involving human research participants and Sex and Gender in Research.

| | |
|---|---|
| Reporting on sex and gender | N/A |
| Population characteristics | N/A |
| Recruitment | N/A |
| Ethics oversight | N/A |

Note that full information on the approval of the study protocol must also be provided in the manuscript.

# Field-specific reporting

Please select the one below that is the best fit for your research. If you are not sure, read the appropriate sections before making your selection.

☒ Life sciences ☐ Behavioural & social sciences ☐ Ecological, evolutionary & environmental sciences

For a reference copy of the document with all sections, see nature.com/documents/nr-reporting-summary-flat.pdf

# Life sciences study design

All studies must disclose on these points even when the disclosure is negative.

| | |
|---|---|
| Sample size | Fig. 1 (n=5), Fig.2 (n=5), Fig.3 (n=5), Extended Data Fig.2 (n=5), Extended Data Fig.3 (n=5), Extended Data Fig.4 (n=5), Extended Data Fig.6 (n=5)<br>Fig. 4, Fig.5, Extended Data Fig.5 (n=6)<br>Sample sizes were determined by the current standard used for mice in immunogenicity experiments based on the minimal amount of mice required to detect significance differences between the groups. Group size were selected based on previous experience with these inbred species. |
| Data exclusions | No data were excluded. |
| Replication | Animal experiments were performed once.<br>Analytical assays were performed at once or twice independently (indicated in the figure legend). |
| Randomization | These animals were inbred and effectively randomized into cages at the arrival to the animal facility. |
| Blinding | No formal blinding was implemented in this study, however the technicians were unaware of sample information when they performed the assays. |

# Reporting for specific materials, systems and methods

We require information from authors about some types of materials, experimental systems and methods used in many studies. Here, indicate whether each material, system or method listed is relevant to your study. If you are not sure if a list item applies to your research, read the appropriate section before selecting a response.

## Materials & experimental systems

| n/a | Involved in the study |
| --- | --- |
| ☐ | ☒ Antibodies |
| ☐ | ☒ Eukaryotic cell lines |
| ☒ | ☐ Palaeontology and archaeology |
| ☐ | ☒ Animals and other organisms |
| ☒ | ☐ Clinical data |
| ☒ | ☐ Dual use research of concern |

## Methods

| n/a | Involved in the study |
| --- | --- |
| ☒ | ☐ ChIP-seq |
| ☐ | ☒ Flow cytometry |
| ☒ | ☐ MRI-based neuroimaging |

# Antibodies

Antibodies used

Western Blotting:
SARS-CoV-2 Spike RBD antibody, rabbit PAb (Sino Biological, 40592-T62, 1:2000 dilution)
anti-rabbit IgG HRP (Santa Cruz, sc-2357, 1:5000 dilution)

ELISA:
mIgGk BP-HRP (Santa Cruz, sc-516102, 1:4000 dilution)
Goat anti-hamster IgG (H+L)-HRP (Southern Biotech, 6060-05, 1:4000 dilution)
Goat anti-human IgG-HRP (Santa Cruz, sc2907, 1:4000 dilution)

Flow Cytometry:
anti-SARS-CoV-2 RBD neutralizing monoclonal antibody (ACRO Biosystems, SPD-M128, 1:200 dilution)
goat anti-human IgG Fc secondary PE (eBioscience, 12-4998-82, 1:33 dilution)
anti-mouse CD107a (clone:1D4B, cat.564347, BD Biosciences, 1:200 dilution)
anti-mouse CD3 (clone:17A2, cat.740268, BD Biosciences, 1:200 dilution)
anti-mouse CD4 (clone:RM4-5, cat.741217, BD Biosciences, 1:400 dilution)
anti-mouse CD8 (clone:53-6.7, cat.612759, BD Biosciences, 1:400 dilution)
anti-mouse CD62L (clone:MEL-14, cat.1044385, Biolegend, 1:200 dilution)
anti-mouse CD44 (clone:IM7, cat.103028, Biolegend, 1:2000 dilution)
anti-mouse CXCR5 (clone:2G8, cat.562856, BD Biosciences, 1:40 dilution)
anti-mouse CXCR3 (clone:CXCR3-173, cat.741681, BD Biosciences, 1:40 dilution)
anti-mouse IFN-g (clone:XMG1.2, cat.505813, Biolegend, 1:400 dilution)
anti-mouse TNF (clone:MP6-XT22, cat.566510, BD Biosciences, 1:100 dilution)
anti-mouse CD154 (clone:MR1, cat.106506, Biolegend, 1:50 dilution)
anti-mouse IL-13 (clone:ebio13A, cat. 25-7133-82, Thermo Fisher Scientific, 1:100 dilution)
anti-mouse IL-21 (clone:FFA21, cat.17-7211-82, Thermo Fisher Scientific, 1:40 dilution)
anti-mouse IL-4 (clone:11B11, cat.564004, BD Biosciences, 1:40 dilution)
anti-mouse IL-2 (clone:JES6-5H4, cat. 503837, Biolegend, 1:200 dilution)
anti-mouse IgM (clone:R6-60.2, cat.550881, BD Biosciences, 1:67 dilution)
anti-mouse B220 (clone:RA3-6B2, cat.553091, BD Biosciences, 1:2857 dilution)
anti-mouse IgD (clone:11-26c.2a, cat.405716, Biolegend, 1:1000 dilution)
anti-mouse CD138 (clone:281-2, cat.563192, BD Biosciences, 1:100 dilution)
anti-mouse NK1.1 (clone:PK136, cat.108733, Biolegend, 1:100 dilution)
anti-mouse CD3 (clone:17A2, cat.100225, Biolegend, 1:100 dilution)
anti-mouse CD38 (clone:90/CD38, cat.740887, BD Biosciences, 1:333 dilution)
anti-mouse CD19 (clone:1D3, cat.612971, BD Biosciences, 1:2000 dilution)
anti-human CD3 (clone:SP34-2, cat.751249, BD Biosciences, 1:100 dilution)
anti-human CD8 (clone:RPA-T8, cat.565695, BD Biosciences, 1:400 dilution)
anti-human CD4 (clone:S3.5, cat.MHCD0418, Thermo Fisher Scientific, 1:50 dilution)
anti-human CD95 (clone:DX2, cat.559773, BD Biosciences, 1:20 dilution)
anti-human CD28 (clone:CD28.2, cat.742037, BD Biosciences, 1:67 dilution)
anti-human CD20 (clone:2H7, cat.745889, BD Biosciences, 1:50 dilution)
anti-human CXCR3 (clone:G025H7, cat.353706, Biolegend, 1:50 dilution)
anti-human CXCR5 (clone:MUSUBEE, cat.61-9185-42, Thermo Fisher Scientific, 1:25 dilution)
anti-human CCR6 (clone:G034E, cat.353443, Biolegend, 1:50 dilution)
anti-human CCR4 (clone:L291H4, cat.359416, Biolegend, 1:50 dilution)
anti-human PD-1 (clone:EH12-2H7, cat.329966, Biolegend, 1:40 dilution)
anti-human CD154 (clone:TRAP1, cat.555699, BD Biosciences, 1:14 dilution)
anti-human IL-4 (clone:8D4-8, cat.560672, BD Biosciences, 1:40 dilution)
anti-human IL-21 (clone:3A3-N21, cat.560493, BD Biosciences, 1:20 dilution)
anti-human IL-13 (clone:JES10-5A2, cat.563580, BD Biosciences, 1:40 dilution)
anti-human IL-17 (clone:BL168, cat.512326, Biolegend, 1:40 dilution)
anti-human TNF (clone:MAb11, cat.502938, Biolegend, 1:50 dilution)
anti-human IFNg (clone:4S.B3, cat.502542, Biolegend, 1:40 dilution)
anti-human IL-2 (clone:MQ-17H12, cat.612836, BD Biolegend, 1:200 dilution)
anti-human IgD (goat polyclonal antibody, cat.2030-09, Southern Biotech, 1:100 dilution)
anti-human IgD (goat polyclonal antibody, cat.2030-02, Southern Biotech, 1:100 dilution)
anti-human CD19 (clone:J3.119, cat.A07770, Beckman coulter, 1:10 dilution)
anti-human CD27 (clone:1A4CD27, cat.6607107, Beckman coulter, 1:25 dilution)
anti-human CD20 (clone:2H7, cat.302314, Biolegend, 1:200 dilution)

anti-human IgM (clone:G20-127, cat.563113, BD Biosciences, 1:100 dilution)
anti-human IgG (clone:G18-145, cat.564230, BD Biosciences, 1:200 dilution)

Validation

SARS-CoV-2 Spike RBD antibody, rabbit PAb (Sino Biological, 40592-T62, 1:2000 dilution) is a specific antibody against SARS-CoV-2 Spike protein RBD domain. No cross-reactivity is detected with Spike protein RBD domain of other coronaviruses, including SARS-CoV, MERS-CoV, HCoV-229E, HCoV-NL63, HCoV-OC43 and HCoV-HKU1.

The antibodies in our study for flow cytometry are all commercially available and their specificity are well characterized by the manufacturers and other users.

# Eukaryotic cell lines

Policy information about cell lines and Sex and Gender in Research

Cell line source(s)

Human embryonic kidney (HEK293T) cells (American Type Culture Collection [ATCC]), Vero kidney epithelial cells (ATCC), VeroE6 (ATCC), and VeroE6 cells constitutively expressing human transmembrane serine protease 2 (TMPRSS2), VeroE6/TMPRSS2 cells (Japanese Collection of Research Bioresources Cell Bank). Expi293 cells were purchased from Thermo Fisher Scientific.

Authentication

not authenticated

Mycoplasma contamination

We confirmed that there is no contamination of Mycoplasma by a PCR kit (Southern Biotech, 13100-01)

Commonly misidentified lines
(See ICLAC register)

No commonly misidentified cell lines are used.

# Animals and other research organisms

Policy information about studies involving animals; ARRIVE guidelines recommended for reporting animal research, and Sex and Gender in Research

Laboratory animals

BALB/c mice (6-8 weeks old), Golden Syrian Hamsters (6-8 weeks old), and adult cynomolgus macaques (6-20 years old)

Wild animals

No wild animals were used in this study

Reporting on sex

BALB/c mice: 5 female per group
Golden Syrian Hamsters: 3 male and 3 female per group
Adult cynomolgus macaques: 2 male and 4 female per group
For mice and hamster studies, we kept the ratio consistent with our previous studies for direct comparison. For non human primate experiment, ratio was determined by the animals availability and male and female were distributed evenly to each groups.

Field-collected samples

No field collected samples were used in this study.

Ethics oversight

The mice and hamster studies were conducted without direct involvement of Quality Assurance but adhered to applicable Noble Life Scienses, Inc (Maryland, US) or Bioqual (Maryland, US) SOPs. This study was conducted in compliance with the current version of the following 1) Animal Welfare Act Regulations (9 CFR); 2) U.S. Public Health Service Office of Laboratory Animal Welfare (OLAW) Policy on Humane Care and Use of Laboratory Animals; 3) Guide for the Care and Use of Laboratory Animals (Institute of Laboratory Animal Resources, Commission on Life Sciences, National Research Council, 1996); and 4) AAALAC accreditation. NHP studies were performed at NIBIOHN with approval from the Committee on the Ethics of Animal Experiments of NIBIOHN (Approved no. #DSR03-25).

Note that full information on the approval of the study protocol must also be provided in the manuscript.

# Flow Cytometry

## Plots

Confirm that:

- [x] The axis labels state the marker and fluorochrome used (e.g. CD4-FITC).
- [x] The axis scales are clearly visible. Include numbers along axes only for bottom left plot of group (a 'group' is an analysis of identical markers).
- [x] All plots are contour plots with outliers or pseudocolor plots.
- [x] A numerical value for number of cells or percentage (with statistics) is provided.

## Methodology

Sample preparation

For analyzing T cells of mice, we performed surface and intracellular cytokine staining of CD4+ and CD8+ T cells. Briefly, splenocytes from mice were incubated in 200 µl RPMI medium containing 10% FBS with or without peptides (17-mers overlapping by 10 residues) corresponding to RBD region or full length of SARS-CoV-2 spike, at a final concentration of 2 µg/ml of each peptide in presence of anti-CD107a (1D4B), for 30 min. Thereafter, 0.2 µl BD GolgiPlug and 0.14 µl BD GolgiStop

(both from BD Biosciences) were added to the cells and the cells were incubated for 5.5 h. The cells were then stained using the LIVE/DEAD™ Fixable Aqua Dead Cell Stain Kit (Thermo Fisher Scientific), and stained with anti-CD3 (17A2), anti-CD4 (RM4-5), anti-CD8 (53-6.7), anti-CD62L (MEL-14), anti-CD44 (IM7), anti-CXCR5 (2G8) and anti-CXCR3 (CXCR3-173) antibodies. After fixation and permeabilization using the Cytofix/Cytoperm kit (BD Biosciences), the cells were stained with anti-IFN-g (XMG1.2), anti-TNF (MP6-XT22), anti-CD154 (MR1), anti-IL-13 (ebio13A), anti-IL-21 (FFA21), anti-IL-4 (11B11) and anti-IL-2 (JES6-5H4) antibodies. The cells were analyzed using a BD FACSymphony A5 flow cytometer (BD Biosciences).

For analyzing of RBD-specific B cells of mice, splenocytes were stained with the LIVE/DEAD Fixable Blue Dead Cell Stain Kit (Thermo Fisher Scientific) for 5 min. Thereafter, the cells were surface-stained with anti-IgM (R6-60.2), anti-B220 (RA3-6B2), anti-IgD (11-26c.2a), anti-CD138 (281-2), anti-NK1.1 (PK136), anti-CD3 (17A2), anti-CD38 (90/CD38) and anti-CD19 (1D3) antibodies for 15 min. Following wash step, the cells were incubated with fluorescent dye-conjugated streptavidin-bound RBD probe for 15 min. The cells were analyzed using a BD FACSymphony A5 flow cytometer (BD Biosciences).

For analyzing antigen-specific T cells of monkeys,we performed surface and intracellular cytokine staining of CD4+ and CD8+ T cells.  Briefly, PBMCs were incubated in 1 ml of RPMI 1640 medium with 50 U/ml benzonase nuclease (Millipore, Darmstadt, Germany), 10% fetal bovine serum and penicillin/streptomycin for 2 h. Next, cells were incubated in medium in the absence or presence of peptides (17-mers overlapping by 10 residues) corresponding to RBD region or full length of SARS-CoV-2 spike at a final concentration of 2 ug/ml for each peptide in presence of anti-CD107a (H4A3), for 30 min.   After that, BD GoldiPlug (BD Biosciences, San Jose, CA) and BD GoldiStop (BD Biosciences, San Jose, CA) were added and incubated for 5.5 h.  Next, the cells were stained with LIVE/DEAD Fixable Blue Dead cell stain (Thermo Fisher Scientific, Waltham, MA) and surface stained with the following: anti-CD3 (SP34-2), anti-CD8 (RPA-T8), anti-CD4 (S3.5), anti-CD95 (DX2), anti-CD28 (CD28.2), anti-CD20 (2H7), anti-CXCR3 (G025H7), anti-CXCR5 (MUSUBEE), anti-CCR6 (G034E), anti-CCR4 (L291H4) and anti-PD-1 (EH12-2H7).  After fix and permeabilization using Cytofix/Cytoperm kit (BD Biosciences, San Jose, CA), cells were stained with anti-CD154 (TRAP1), anti-IL-4 (8D4-8), anti-IL-21 (3A3-N21), anti-IL-13 (JES10-5A2), anti-IL-17 (BL168), anti-TNF (MAb11), anti-IFNg (4S.B3) and anti-IL-2 (MQ-17H12).

For analyzing antigen-specific B cells of monkeys, peripheral blood mononuclear cells and lymph node cells were stained with the LIVE/DEAD Fixable Blue Dead Cell Stain Kit (Thermo Fisher Scientific) for 5 min. Thereafter, the cells were surface-stained with anti-IgD (goat polyclonal antibody), anti-CD19 (J3.119), anti-CD27 (1A4CD27), anti-CD20 (2H7), anti-IgM (G20-127), anti-IgG (G18-145) and anti-CD3 (SP34-2) antibodies for 15 min. Following wash step, the cells were incubated with fluorescent dye-bound RBD probe for 15 min. The cells were analyzed using a BD FACSymphony A5 flow cytometer (BD Biosciences).

Instrument

BD FACSymphony A5 flow cytometer (BD Biosciences)

Software

FlowJo Software version 10.8.1.

Cell population abundance

Cells were not enriched prior to in vitro stimulation.

Gating strategy

For analyzing T cells in mice: After gating live single T cells based on forward scatter area and height (FSC-A and -H), side scatter area (SSC-A), live/dead cell exclusion, and CD3 staining, we separated the cells into CD4 T cells and CD8 T cells. Subsequently, CD4 and CD8 T cells were further divided into memory phenotypes based on the expression of CD62L and CD44. We defined CD4 memory T cells expressing IFN-g, TNF or IL-2 as Th1 cells and expressing IL4 or IL-13 as Th2 cells.

For analyzing B cells in mice: After gating live single B cells based on FSC-A and -H, SSC-A, live/dead cell exclusion, CD3 and NK1.1 positive cell exclusion, and CD19 and B220 staining, we further gated the B cells into CD138-IgM-IgD-CD38+memory B cells. RBD probe-binding cells in memory B cells were defined as RBD-specific B cells.

For analyzing T cells in NHP: After gating live single T cells, based on FSC-A and -H, SSC-A, live/dead cell exclusion, and CD3 staining, we separated the cells into CD4 and CD8 T cells. Subsequently, CD4 and CD8 T cells were further divided into memory phenotypes based on the expression of CD28 and CD95. For spike-specific CD4 T cells, memory cells were gated based on expression of CD154. We defined CD154+CD4 T cells expressing IFN-g, TNF or IL-2, or CXCR3 as Th1 cells, expressing IL-4 or IL-13, or CCR4 as Th2 cells, expressing IL-17 or CCR6 as Th17 cells and expressing CXCR5 and PD-1, or IL-21 as Tfh cells.

For analyzing B cells in NHP: After gating live single B cells, based on FSC-A and -H, SSC-A, live/dead cell exclusion, CD3 positive cell exclusion, and CD19 and CD20 staining, we further gated the B cells into IgM-IgD-IgG+CD27+memory B cells. RBD probe-binding cells in memory B cells were defined as RBD-specific B cells.

☒ Tick this box to confirm that a figure exemplifying the gating strategy is provided in the Supplementary Information.
